# Innovative determination of phytohormones in *Aloe vera*

**DOI:** 10.3389/fchem.2024.1490639

**Published:** 2025-01-20

**Authors:** Muhammad K. Hakeem, Meera Maraqa, Sampath K. Elangovan, Esam Eldin Saeed, Ajay Kumar Mishra, Khaled M. Hazzouri, Iltaf Shah, Khaled M. A. Amiri

**Affiliations:** ^1^ Department of Chemistry, College of Science, United Arab Emirates University (UAEU), Al Ain, United Arab Emirates; ^2^ Khalifa Center for Genetic Engineering and Biotechnology, United Arab Emirates University, Al Ain, United Arab Emirates

**Keywords:** LC-MS/MS, Aloe vera, phytohormones, liquid chromatography mass spectrometry, plant hormone, method validation

## Abstract

**Introduction:**

Aloe vera is widely known for its therapeutic properties, but concerns regarding the levels of phytohormones and their potential impact on human health highlight the need for advanced analytical techniques. This study aims to develop and validate a sensitive method for the determination of six key phytohormones in Aloe vera using Liquid Chromatography-Tandem Mass Spectrometry (LC-MS/MS).

**Methods:**

A validated LC-MS/MS method was optimized for the determination and quantification of six phytohormones in Aloe vera: Abscisic Acid (ABA), Salicylic Acid (SA), Indole-3-Acetic Acid (I3AA), Gibberellic Acid (GA), 6-Benzylaminopurine (6BAP), and Isopentenyladenine (ISA). The sample extraction process and mobile phase composition were optimized to enhance chromatographic separation and mass spectrometry sensitivity. A C-18 column was used for separation, and a triple quadrupole mass spectrometer was employed for quantification. The method’s performance was assessed in terms of linearity, sensitivity, and limits of detection.

**Results:**

The LC-MS/MS method exhibited excellent linearity (*R* ^2^ > 0.99) and low limits of detection for all six phytohormones. Four of the six analytes were identified as predominant in Aloe vera. Quantitative analysis showed that ABA was the most abundant phytohormone, with a median concentration of 8.39 ng/mL, followed by I3AA (4.32 ng/mL), SA (3.16 ng/mL), and GA (1.55 ng/mL).

**Discussion:**

This study provides a comprehensive and validated LC-MS/MS method for profiling phytohormones in Aloe vera. The results underscore the significant role of ABA, I3AA, SA, and GA in the plant's hormonal profile, offering a valuable tool for the analysis of phytohormonal content in Aloe vera and other plant species. The method is particularly beneficial for addressing health-related concerns regarding the presence and concentration of phytohormones in Aloe vera.

## Introduction

Phytohormones are natural compounds that are found in trace levels in plants. Their presence is essential in maintaining plant growth and maturation, regulating the division of cells and the differentiation of tissues, as well as controlling plant behavior because of various stimuli (Bhatt et al., 2020; Zhao et al., 2021; Pal et al., 2023; Asif et al., 2022). Phytohormones are characterized by their diverse chemical properties, but they can be poorly stable upon exposure to light or heat (Jha et al., 2022). Based on their structures and physiological functions, phytohormones are classified into six main types, entailing Auxins (Indole derived), Cytokinins (Adenine derived), Abscisic Acid (Carotenoids derived), Gibberellin (Terpene derived), Ethylene (gas derived), and Brassinosteroids (Bhatt et al., 2020; Tariq et al., 2022; Bhattacharya, 2022). Within each category, there could be several compounds with similar physiological functions. For example, Indole-3-Acetic Acid (I3AA) and 2-Naphthalene Acetic Acid (2NAA) are auxins that promote plant development in different ways, with the former promoting growth through the excessive production of roots hairs and lateral roots (Zhang et al., 2022; Etesami and Glick, 2024), while the latter is involved in the vegetative propagation of cuts in the stems or leaves (Ashok and Ravivarman, 2020; Kaviani et al., 2023). Cytokinins such as Isopentenyl Adenine Indole and 6-Benzylaminopurine play a pivotal role in regulating myriad processes associated with plant development and the cell cycle (Mangena, 2022; Butt and Gul, 2023). In addition, Abscisic Acid is a key signaling molecule in responding to a range of stimuli, involving abiotic and biotic stresses (Bharath et al., 2021; Muhammad Aslam et al., 2022). Gibberellic Acid, on the other hand, is mainly involved in activating the developmental switches in the different stages of a plant’s life cycle (i.e., development from the vegetative phase to the reproductive phase) (Hernández-García et al., 2021; Katyayini et al., 2020). Moreover, Salicylic Acid not only contributes to the plant’s growth but is crucial in prompting certain responses associated with plant defense (Zhong et al., 2021; Arif et al., 2020).

Aloe vera, a perennial succulent plant renowned for its diverse therapeutic properties, has captured global attention and witnessed a surge in demand within the herbal products market. It is a widely used plant that belongs to the Liliaceae family. Due to its anti-inflammatory and antimicrobial properties, Aloe vera has been utilized in traditional medicine to heal skin injuries and digestive issues (Adlakha et al., 2022; Gulati et al., 2021). Moreover, recent studies suggest its potential as an antitumor agent, further expanding its therapeutic applications (Sinha et al., 2023; Ali et al., 2020). Such therapeutic properties have advocated its extensive integration in both the pharmaceutical and the food industry (Martínez-Sánchez et al., 2020). With over 500 species of the aloe genus, only 10 species are considered of medicinal value, among which Aloe barbadensis miller is considered the most biologically active species (Leitgeb et al., 2021; Bista et al., 2020; Palaniyappan et al., 2023). The widespread use of Aloe vera in various commercial products, including cosmetics, dietary supplements, and pharmaceuticals, underscores the need for a comprehensive understanding of its chemical composition. Aloe vera’s therapeutic potential is attributed to its rich chemical diversity, including polysaccharides, phenolic compounds, and bioactive molecules (Pradhan, 2023; Rajesh et al., 2023). The identification and quantification of phytohormones within Aloe vera are crucial for unraveling the intricate relationship between its chemical profile and therapeutic effects.

Phytohormones, while essential for plant physiology, have been shown to influence various physiological processes in mammals as well. For instance, compounds like salicylic acid, gibberellic acid, indole-3-acetic acid, and abscisic acid, which are found in Aloe vera, may interact with mammalian hormonal pathways, potentially affecting immune responses, metabolism, and even cancer progression (Choi and Chung, 2003; Maan et al., 2018). For instance, ABA has been shown to modulate glucose metabolism and exhibit anti-inflammatory properties, offering potential therapeutic implications for managing metabolic disorders (Gharib et al., 2024). Similarly, SA is widely recognized for its anti-inflammatory and immune-modulatory effects, which may contribute to human health when consumed through plant-based products (Thorat et al., 2023). However, excessive or imbalanced exposure to phytohormones, such as SA, could disrupt hormonal homeostasis or trigger adverse reactions, emphasizing the need for accurate and controlled quantification (Ali et al., 2024). I3AA, an auxin, has been investigated for its potential anticancer properties, with studies suggesting it may induce apoptosis in certain cancer cell lines (Phytohormones as Potential Anticancer Agents, 2024; Mukherjee et al., 2022). Meanwhile, GA has been reported to interact with mammalian signaling pathways, though its precise effects remain underexplored and warrant further investigation (Castro-Camba et al., 2022). This raises important questions about the safety and health consequences of phytohormones in Aloe vera when used in consumer products. Given the increasing incorporation of Aloe vera into products intended for human use, it is crucial to investigate its phytohormonal composition to understand the potential effects on human health. This study aims to fill this gap by systematically identifying and quantifying key phytohormones within Aloe vera, thereby providing a scientific foundation for evaluating its safety and efficacy. These findings can contribute to the development of regulatory standards and guidelines, ensuring the safety and effectiveness of Aloe vera products for consumers.

The determination of phytohormones is challenged by the extremely low concentration of these compounds in the plant tissues along with the varied concentration ranges among different plant species. Traditional methods for detecting phytohormones in plant extracts often fall short in terms of sensitivity, selectivity, and precision. In the early stage, bioassays and immunoassays were the common methods used for the determination of phytohormones (Wang et al., 2020; Jiang et al., 2020). However, these methods have been phased out due to their low sensitivity, specificity, and inability to simultaneously detect multiple plant hormones. For example, bioassays often lack the resolution required for distinguishing structurally similar compounds, while immunoassays are limited by cross-reactivity and low multiplexing capabilities. Similarly, HPLC-UV methods, while useful for single-analyte determination, fail to provide adequate sensitivity and selectivity for simultaneous analysis of diverse phytohormones in complex matrices. The inadequacies of these methods underscore the urgency of adopting advanced analytical techniques to provide more accurate and reliable results. Significant progress in the determination of phytohormones has been witnessed in the last decade due to the advancement in the field of chromatography and mass spectroscopy (Wang et al., 2020; Recent Advances in the Chromatographic). Specifically, the integration of Liquid Chromatography-Tandem Mass Spectrometry (LC-MS/MS) emerges as a cutting-edge solution, offering unparalleled capabilities in the identification and quantification of phytohormones (Wang et al., 2020; Bisht et al., 2021; Vaidya et al., 2023; Hakeem et al., 2024; Ashraf et al., 2024). LC-MS/MS surpasses traditional approaches by enabling simultaneous determination of multiple analytes with high sensitivity and specificity, even at trace levels. Furthermore, its ability to handle complex matrices makes it particularly suitable for studying the phytohormonal profile of Aloe vera, where bioactive compounds exist in minute concentrations alongside numerous interfering substances.

However, information about the levels of phytohormones in many other plant species including Aloe vera is still limited. This gap hinders the establishment of comprehensive regulatory frameworks for herbal products, prompting the need for rigorous scientific inquiry to bridge this knowledge gap. In this context, our research addresses these critical gaps by presenting an innovative and robust LC-MS/MS methodology to detect and quantify phytohormones in Aloe vera. The developed method evaluates phytohormone levels in Aloe vera, adding to existing knowledge about their concentrations in other plants. These results may also serve as reference values for decision-making about the use of Aloe vera in commercial products. This breakthrough promises to enhance consumer safety, refine regulatory standards, and contribute significantly to the scientific understanding of Aloe vera’s therapeutic composition.

## Method and materials

### Chemical reagents and standards

Abscisic Acid (ABA) 98%, Gibberellic Acid (GA) 90%, Indole-3-Acetic Acid (I3AA) 100%, 6-BenzylAminoPurine (6BAP) 99%, Isopentenyl Adenine (ISA) 98.5%, 2-Naphthalene Acetic Acid (2NAA) 95%, Salicylic Acid (SA) 99%, and Salicylic Acid D4 (SA-D4) (internal standard) were purchased from Sigma Aldrich (USA). LC-MS grade methanol, formic acid and acetic acid (LC-MS grade) were obtained from Supelco (Germany), Fluka (Switzerland). Milli-Q-Water obtained from in-house (UAE university).

### Preparation of standard solution

Individual phytohormone standards, each weighing approximately 5.00 g, were carefully measured and dissolved in 5.00 mL of LC-MS/MS grade methanol in a volumetric flask. This resulted in a stock solution with a concentration of 1.00 mg/ml. A blend of all individual standard stock solutions was prepared using a diluent consisting of a 50:50 methanol-water to prepare an intermediate solution. This intermediate solution was then used to generate calibration standard solutions, crucial for the linearity step during the analysis. The deliberate use of methanol as the solvent ensured optimal solubility and stability of the phytohormones in the solution. This not only laid the groundwork for a reliable standard against which samples could be measured but also upheld the integrity of the phytohormones throughout the analysis.

### Sample preparation and extraction of phytohormones

The quantification of phytohormones in Aloe vera was conducted with a focus on ensuring accuracy and precision throughout the analytical protocol. To achieve this, 13 Aloe vera samples were randomly selected from different sources to represent natural variability within the species. These samples were subjected to consistent environmental conditions to minimize external factors influencing phytohormone concentrations. The observed variations in phytohormone levels among these samples are indicative of the natural variability expected within a broader population. This approach allowed for a robust assessment of phytohormonal content, ensuring that the analytical results are reflective of the typical range of phytohormones found in Aloe vera plants. The samples were thoroughly washed with DI water and left to dry under ambient conditions before collecting the gel. The protocol used for sample preparation was developed by the authors, drawing on methodologies from existing literature for phytohormone analysis in plant matrices. Studies have demonstrated the efficacy of acetonitrile-based extraction solutions and acidified solvents for stabilizing phytohormones in complex plant matrices (Sutcharitchan et al., 2020). Preliminary experiments were conducted to adapt parameters such as solvent composition, sample-to-solvent ratio, and centrifugation speed to optimize analyte recovery and stability. The chosen conditions, including the use of 1% acetic acid in acetonitrile as the extraction solvent, were shown to yield consistent recoveries exceeding 95% for all target analytes. These adjustments ensured the protocol was robust and tailored specifically to Aloe vera phytohormone analysis.

The extraction process commenced with weighing 1.00 g of Aloe vera gel. To enhance accuracy, 0.05 mL of salicylic acid D4 (500.00 ng/mL), was added as an internal standard. Salicylic acid D4 was selected due to its structural and physicochemical similarity to the target phytohormones, which allows it to mimic their behavior during chromatographic separation and mass spectrometric determination. The addition of the internal standard was thoroughly optimized to ensure consistent recovery and reliable quantification across all analytes. The samples were thoroughly vortexed to ensure homogeneity. Subsequently, a solution comprising 5.00 mL with 1% acetic acid in acetonitrile was added to the sample and vortexed to ensure thorough mixing. To separate the components, the resulting mixture underwent centrifugation at 4,500 rpm for 10 min. Following centrifugation, the supernatant was meticulously filtered through a 0.45 µm filter and extracted for further analysis. The prepared solution was then injected into an LC-MS/MS instrument for a detailed analysis of the sample. The entire procedure was performed in triplicate for each Aloe vera sample, providing a robust assessment of the developed analytical protocol’s precision and reliability.

### LC-MS/MS analysis and method development

LC-MS/MS analysis was carried out using Shimadzu (Japan) model LC-30AD (Nexera X2) binary pump and LCMS-8060 Shimadzu (Japan). The chromatographic separation was performed using Zorbax Eclipse Plus C18 (4.6 × 100 mm, 3.5 µm pore size) column sourced from Agilent Technologies. The mobile phase consisted of 0.01% formic acid (v/v) in a mixture of water and methanol (35:65 v/v). A 10.0 µL volume of the sample was injected, and the mobile phase flowed at a rate of 0.50 mL/min. The oven temperature was set at a constant 30°C during the analysis. The LC system was connected to a triple quadrupole mass spectrometer detector. Phytohormones underwent ionization in both positive and negative electrospray ionization (ESI) modes. Quantification was achieved through the multiple reaction monitoring (MRM) mode. In the optimization of the LC-MS/MS method, a series of standards were employed, and the resulting Multiple Reaction Monitoring (MRM) data underwent comprehensive analysis. The selection of optimal parent and daughter ions was a pivotal step in refining the method. The collision energy (CE) was carefully fine-tuned based on the relative abundance of the parent and daughter ions, as detailed in Table 1. Q1 and Q3 in Table 1 indicate the mass parent and mass product ion respectively. A detailed description of Table 1 can be found under the Supplementary Information (SI) section.

**TABLE 1:** Qualification and quantification of MRM transition for phytohormones using LC-MS/MS.

Compound	Q1 (m/z)	Q3 (m/z)	Dwell time (ms)	Q1 pre-Bias (V)	CE (V)	Q3 pre-Bias	Ionization mode
I3AA	176.00	130.10	100	−17	−15	−22	Positive
	176.00	77.10	100	−16	−42	−15	
	176.00	103.05	100	−16	−29	−23	
2NAA	184.90	117.05	100	22	11	26	Negative
	184.90	100.05	100	20	34	38	
	184.90	141.10	100	22	10	20	
6BAP	224.05	133.15	100	24	23	26	Negative
	224.05	132.15	100	24	32	26	
	224.05	188.00	100	26	13	22	
GA	345.25	143.20	100	17	29	13	Negative
	345.25	239.20	100	17	16	30	
	345.25	221.35	100	17	25	22	
SA	137.20	93.15	100	13	21	11	Negative
	137.20	65.20	100	14	28	15	
	137.20	100.00	100	15	27	26	
ABA	263.10	153.25	100	28	12	20	Negative
	263.10	219.25	100	28	14	24	
	263.10	204.30	100	28	20	26	
SA-D4	141.10	97.10	100	14	21	12	Negative
	141.10	59.10	100	14	12	14	

Q1 pre-bias: Voltage needed to promote ionization of precursor ion, Q3 pre-bias: Voltage needed to promote ionization of product ion).

To establish the instrument’s detection limits for each phytohormone, a rigorous approach was employed. Three replicates of each sample were analyzed, and the signals from each sample at the retention time of the respective phytohormone were recorded. The standard deviation of these signals was calculated. Utilizing the slope ratio between the internal standard and each phytohormone, the standard deviation values were then applied to determine the limit of detection (LOD). This method not only ensures precision in quantification but also provides a robust assessment of the sensitivity of the LC-MS/MS system in detecting trace amounts of phytohormones in Aloe vera samples.

### Method validation

The validation of the testing method is crucial to determine its reliability and ability to generate accurate and valuable data. This meticulous process ensures that the analytical procedure consistently delivers results within defined parameters. The criteria employed for validation encompass selectivity, sensitivity, precision, accuracy, linearity, and matrix effect (Cheng et al., 2022; Wille et al., 2022; Williams et al., 2023). The matrix effect was evaluated to ensure that the Aloe vera matrix did not interfere significantly with analyte determination or quantification. Sensitivity is expressed through the limit of detection (LOD) and the limit of quantitation (LOQ). The LOD represents the lowest concentration of an analyte that can be reliably distinguished from background noise, while the LOQ is the lowest concentration that can be quantified with acceptable precision and accuracy. Both parameters were calculated using the standard deviation of the response (SD) and the slope of the calibration curve (S) as per modern guidelines (Rajmane Akash and Shinde Komal (2023)). Precision, the degree of scatter among measurements, and accuracy, the proximity of test values to actual values, were precisely evaluated. Three quality control (QC) samples (High, medium, and low) concentrations were used to evaluate both intra- and inter-day accuracy and precision. These parameters collectively ensure the consistency and reliability of the analytical method. The detailed results of the validation process are presented in Table 2. This validation demonstrates the sensitivity and robustness of the developed analytical protocol.

**TABLE 2:** Applied quality control measures in the determination of the target phytohormones.

Compound	Quality control (QC)	Conc. (ng/mL)	Linearity Range (ng/mL)	R^2^	LOD (ng/mL)	LOQ (ng/mL)	Intraday analysis		Interday analysis	
							Accuracy (%)	RSD (%)	Accuracy (%)	RSD (%)
ASA	LOQ	0.99	0.99–203.81	0.99	0.40	1.20	105.97	10.08	101.77	6.78
	2×LOQ	1.99					103.82	4.74	100.08	6.33
	5×LOQ	4.99					100.15	2.34	98.77	5.24
GA	LOQ	2.02	2.02–411.26	0.99	0.33	1.01	90.53	4.92	87.73	14.04
	2×LOQ	4.03					90.83	9.18	97.80	8.39
	5×LOQ	10.08					95.64	3.14	95.88	4.89
I3AA	LOQ	2.07	2.07–422.40	0.98	0.27	0.83	89.96	3.71	101.66	3.78
	2×LOQ	4.14					107.12	2.02	108.37	2.69
	5×LOQ	10.35					111.34	3.44	112.51	1.68
6BAP	LOQ	2.56	2.56–522.72	0.99	0.51	1.54	84.59	7.54	83.85	10.89
	2×LOQ	5.12					97.22	12.2	95.87	4.60
	5×LOQ	12.81					100.28	1.86	97.79	1.81
ISA	LOQ	0.16	0.16–32.78	0.99	0.01	0.04	95.65	2.11	99.28	5.11
	2×LOQ	0.32					116.25	4.38	116.61	1.24
	5×LOQ	0.80					96.68	3.63	98.09	1.60
2NAA	LOQ	126.62	126.62–25840.0	0.99	36.40	110.30	101.67	4.55	105.70	9.28
	2×LOQ	253.23					100.53	1.83	104.06	5.67
	5×LOQ	633.08					101.66	2.27	104.10	2.92
SA	LOQ	1.06	1.06–216.22	0.99	0.54	1.62	90.43	14.68	110.78	9.59
	2×LOQ	2.12					89.45	9.51	99.36	5.82
	5×LOQ	5.30					86.13	4.74	94.57	3.17

The results from Table 2 collectively demonstrate the method’s ability to meet quality control measures, providing a foundation for accurate and reliable determination of target phytohormones in Aloe vera samples. A detailed description of Table 2 can be found in Supplementary Information (SI) section. These findings not only validate the analytical approach but also underscore its applicability in ensuring the quality and safety of Aloe vera products for consumers.

## Results and discussion

### Validation results

**FIGURE 1:**
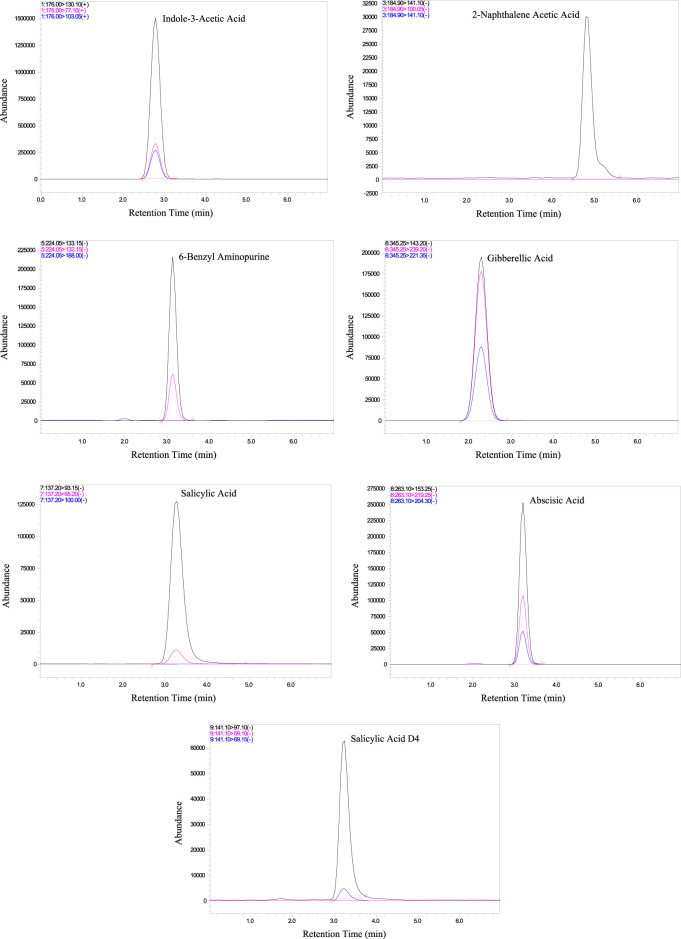
Chromatograms of the target phytohormones and internal standard.

The analytical method developed for phytohormone analysis was meticulously validated and applied to Aloe vera samples. The validation process evaluated the method’s selectivity, sensitivity, matrix effect, precision, and accuracy to ensure its suitability for the analysis. Matrix Effect was assessed by comparing the responses of analytes spiked into blank extracts with those spiked into pure solvent. The observed signal suppression or enhancement for all six phytohormones was within the acceptable range (80%–120%), indicating minimal interference from the Aloe vera matrix (Table 2). Further validation of the analytical method was assessed by evaluating the retention times and intensity of phytohormones. The results affirm the establishment of a robust linear relationship among the analytes, as evidenced by an exceptional regression value (*R* ^2^ = 0.99) (Supplementary Figure S1). This high degree of correlation between phytohormone concentration and signal intensity is paramount for ensuring precise and reliable quantification across a diverse concentration range. To visually represent the efficacy of the method, Figure 1 presents chromatograms depicting the chromatographic profiles of the six target phytohormones under optimal conditions. Each hormone manifests as a distinct peak at its specific retention time, demonstrating the method’s ability to precisely separate and identify individual compounds. The clarity and separation of these peaks are essential for accurate quantification, ensuring that each phytohormone is distinctly identified and measured.

The chromatograms (Figure 1) reveal sharp, well-resolved peaks for all phytohormones, underscoring the method’s ability to selectively quantify the target. The clarity and distinctiveness of these peaks contribute to the method’s selectivity, reinforcing its efficacy in phytohormone determination. The chromatographic results not only showcase the robustness of the method but also lay the foundation for accurate quantification, thereby enhancing the reliability of the analytical approach for phytohormone analysis in plant samples.

### Sample analysis

**FIGURE 2:**
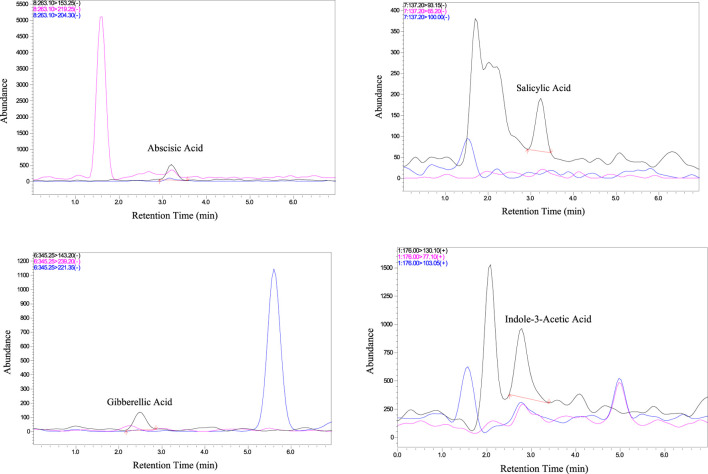
Chromatograms of the target phytohormones in real sample.

Following the successful validation of the LC-MS/MS method, it was applied to the analysis of phytohormones in Aloe vera samples. Upon analysis, it was observed that out of the six targeted phytohormones, four were successfully detected using the developed LC-MS/MS method. Figure 2 shows the chromatograms of real sample of Aloe vera highlighting the unique chemical properties of each phytohormone in terms of variations in their retention times. The observed differences in retention times, as depicted in Figure 2, offer a valuable visual tool for researchers to easily identify and distinguish individual phytohormones. This critical information contributes to the understanding of hormonal dynamics within Aloe vera, paving the way for further investigations into its therapeutic potential and adaptive responses.

**FIGURE 3:**
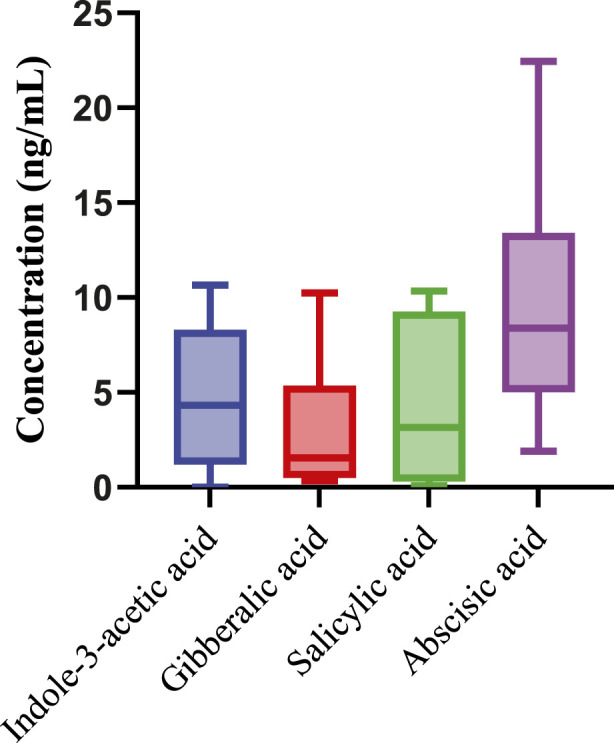
Box and Whisker plot showing concentration ranges (ng/mL) of detected phytohormones in the Aloe vera samples (N = 13).

The variability in concentrations among different samples highlights the heterogeneity in phytohormone composition within Aloe vera, which could be influenced by various factors such as plant age, environmental conditions, and cultivation practices. The observed concentrations provide valuable insights into the phytohormone profile of Aloe vera, contributing to a deeper understanding of its physiological and therapeutic properties. Further exploration of these variations may uncover implications for the quality and efficacy of Aloe vera products in diverse applications. In Figure 3, the box-and-whisker plots illustrate the concentrations of phytohormones in the tested Aloe vera samples, providing a comprehensive overview of overall phytohormone profile.

ABA consistently shows the highest median concentration across all samples, highlighting its dominant presence in the phytohormone composition of Aloe vera. I3AA follows, with the second-highest median concentration, underscoring its significant role in the samples. SA and GA rank third and fourth, respectively, in terms of median concentrations. The distribution and spread of the data, as depicted by the whiskers and interquartile ranges, reveal the variability within each phytohormone group. Despite these variations in median values, statistical analysis confirms that the differences in concentrations among the phytohormones are not statistically significant at a 95% confidence level. This conclusion is supported by the overlapping confidence intervals and similar interquartile ranges, suggesting that the observed differences fall within the normal range of variation.

This study presents a novel LC-MS/MS methodology for the comprehensive analysis of phytohormones in Aloe vera, overcoming limitations of previous approaches that predominantly focused on single analytes or employed less sensitive techniques. The optimized extraction and analytical protocols enable precise and reproducible quantification of key phytohormones, filling critical gaps in the literature regarding Aloe vera’s chemical profile. By providing a detailed understanding of the phytohormonal composition, this work establishes a robust foundation for evaluating Aloe vera’s therapeutic potential and safety in consumer products. These findings also highlight the complex and dynamic regulation of phytohormones in Aloe vera, emphasizing the need for further research into their roles and interactions. The methodology developed in this study provides a reliable framework for future investigations, enabling deeper insights into the biological and therapeutic significance of phytohormones in Aloe vera and other plant systems. The validated LC-MS/MS method demonstrates exceptional robustness, with the capacity to separate, detect, and quantify phytohormones with high sensitivity and selectivity. These attributes underscore its potential adaptability to other plant species, particularly those with similarly intricate biochemical compositions. The use of acidified acetonitrile as an extraction solvent and the optimized chromatographic conditions are broadly applicable to other plants with diverse phytohormonal profiles. For example, this protocol could be extended to other medicinal plants such as *Echinacea purpurea* or *Camellia sinensis*, where phytohormone analysis is crucial for understanding their therapeutic properties. Furthermore, the method’s ability to analyze trace phytohormones makes it suitable for high-throughput studies in agricultural research, where the hormonal regulation of growth and stress responses across different crops is a key area of interest.

## Conclusion

In the current study, we have successfully developed, optimized, and validated a robust LC-MS/MS method for the accurate determination of phytohormone concentrations in Aloe vera samples to advance our understanding of the intricate signaling mechanisms within this medicinal plant. The optimization of the LC-MS/MS method involved the systematic variation of phase composition, leading to enhanced separation and resolution of phytohormones within the complex matrix of Aloe vera. Following the guidelines established by regulatory authorities, such as the US Food and Drug Administration (FDA), our validation process rigorously examined various parameters, demonstrating the method’s reliability and robustness. Compared to traditional techniques such as bioassays, immunoassays, and HPLC-UV, the LC-MS/MS method provides significant advancements in sensitivity, selectivity, and throughput. The LC-MS/MS method, with its high sensitivity (LOD as low as 0.01 ng/mL) and multiplexing capabilities, addresses these limitations and sets a new benchmark for phytohormone analysis. Our results indicate that ABA consistently emerged as the predominant hormone, followed by SA, I3AA, and GA. Variations in concentrations were observed among different samples, showcasing the heterogeneous nature of phytohormone composition in Aloe vera. Our findings stimulate further exploration, inviting researchers to delve into the world of phytohormones and their role in shaping the unique characteristics of different plants. Our findings reveal a distinct distribution of phytohormones, with ABA and SA predominating, suggesting their pivotal roles in the plant’s stress response and defense mechanisms, and hint at potential gender-specific hormonal regulation. This detailed interpretation of results not only highlights the method’s effectiveness but also opens new avenues for research into the physiological roles of phytohormones in Aloe vera and other medicinal plants. By establishing baseline levels of phytohormones, our study contributes to the safety evaluation of Aloe vera-based products, correlating hormonal profiles with therapeutic effects and supporting the traditional and potential new uses of Aloe vera in pharmaceuticals and nutraceuticals, thus benefiting both scientific research and consumer wellbeing.

## Data Availability

The original contributions presented in the study are included in the article/Supplementary Material, further inquiries can be directed to the corresponding author.

## Abbreviations

I3AA, Indole-3-Acetic Acid; 6BAP, 6-BenzylAminoPurine; GA, Gibberellic Acid; ISA, Isopentenyl Adenine; SA, Salicylic Acid; 2NAA, 2-Naphthalene Acetic Acid; ABA, Abscisic Acid; ng/mL, nanogram/millilitre.

## References

[B1] Adlakha K. Koul B. Kumar A. (2022). Value-added products of Aloe species: panacea to several maladies. South Afr. J. Bot. 147, 1124–1135. 10.1016/j.sajb.2020.12.025

[B2] Ali A. Kant K. Kaur N. Gupta S. Jindal P. Gill S. S. (2024). Salicylic acid: homeostasis, signalling and phytohormone crosstalk in plants under environmental challenges. South Afr. J. Bot. 169, 314–335. 10.1016/j.sajb.2024.04.012

[B3] Ali K. Saquib Q. Siddiqui M. A. Ahmad J. Al-Khedhairy A. A. Musarrat J. (2020). Anti-cancer efficacy of Aloe vera capped hematite nanoparticles in human breast cancer (MCF-7) cells. J. Drug Deliv. Sci. Technol. 60, 102052. 10.1016/j.jddst.2020.102052

[B4] Arif Y. Sami F. Siddiqui H. Bajguz A. Hayat S. (2020). Salicylic acid in relation to other phytohormones in plant: a study towards physiology and signal transduction under challenging environment. Environ. Exp. Bot. 175, 104040. 10.1016/j.envexpbot.2020.104040

[B5] Ashok A. Ravivarman J. (2020). Efficacy of naphthalene acetic acid on root promotion on vegetative propagation of Tecoma stans under mist chamber of semi-arid tropic region. J. Pharmacogn. Phytochem. 9 (4), 3000–3002.

[B6] Asif R. Yasmin R. Mustafa M. (2022). Phytohormones as plant growth regulators and safe protectors against biotic and abiotic stress. Plant Horm Recent Adv New Perspect Appl, 115–130.

[B37] Ashraf D. Morsi R. Usman M. Meetani M. A. (2024). Recent Advances in the Chromatographic Analysis of Emerging Pollutants in Dairy Milk: A Review (2018–2023). Molecules. 29 (6), 1296. 10.3390/molecules29061296 38542932 PMC10974215

[B8] Bharath P. Gahir S. Raghavendra A. S. (2021). Abscisic acid-induced stomatal closure: an important component of plant defense against abiotic and biotic stress. Front. Plant Sci. 12, 615114. 10.3389/fpls.2021.615114 33746999 PMC7969522

[B9] Bhatt D. Nath M. Sharma M. Bhatt M. D. Bisht D. S. Butani N. V. (2020). “Role of growth regulators and phytohormones in overcoming environmental stress,” in Prot chem agents amelior plant abiotic stress biochem mol perspect, 254–279.

[B10] Bhattacharya A. (2022). “Plant growth hormones in plants under low-temperature stress: a Review,” in Physiol process plants low temp stress, 517–627.

[B11] Bisht N. Gupta A. Awasthi P. Goel A. Chandran D. Sharma N. (2021). Development of a rapid LC-MS/MS method for the simultaneous quantification of various flavonoids, isoflavonoids, and phytohormones extracted from Medicago truncatula leaves. J. Liq. Chromatogr. Relat. Technol. 44 (15-16), 776–787. 10.1080/10826076.2022.2040028

[B12] Bista R. Ghimire A. Subedi S. (2020). Phytochemicals and antioxidant activities of aloe vera (aloe barbadensis). J. Nut Sci. Heal Diet. 1 (1), 25–36. 10.47890/jnshd/2020/rbista/10243803

[B13] Butt R. S. Gul A. (2023). “Induction of physiological and metabolic changes in plants by plant growth regulators,” in Phytohormones and stress responsive secondary metabolites (Elsevier), 141–159.

[B14] Castro-Camba R. Sánchez C. Vidal N. Vielba J. M. (2022). Interactions of gibberellins with phytohormones and their role in stress responses. Horticulturae 8 (3), 241. 10.3390/horticulturae8030241

[B15] Cheng W. L. Markus C. Lim C. Y. Tan R. Z. Sethi S. K. Loh T. P. (2022). Calibration practices in clinical mass spectrometry: review and recommendations. Ann. Lab. Med. 43 (1), 5–18. 10.3343/alm.2023.43.1.5 36045052 PMC9467832

[B16] Choi S. Chung M. H. (2003). A review on the relationship between Aloe vera components and their biologic effects. Seminars Integr. Med. 1, 53–62. 10.1016/s1543-1150(03)00005-x

[B17] Etesami H. Glick B. R. (2024). Bacterial indole-3-acetic acid: a key regulator for plant growth, plant-microbe interactions, and agricultural adaptive resilience. Microbiol. Res. 281, 127602. 10.1016/j.micres.2024.127602 38228017

[B18] Gharib A. Marquez C. Meseguer-Beltran M. Sanchez-Sarasua S. Sanchez-Perez A. M. (2024). Abscisic acid, an evolutionary conserved hormone: biosynthesis, therapeutic and diagnostic applications in mammals. Biochem. Pharmacol. 229, 116521. 10.1016/j.bcp.2024.116521 39251140

[B19] Gulati P. College M. Sahib F. (2021). A review on medicinal properties of Aloe vera plant and it’s profile. Int. Res. J. Mod. Eng. Technol. Sci. 5, 22.

[B20] Hakeem M. K. Elangovan S. Rafi M. George S. Shah I. Amiri K. M. A. (2024). Advancing antibiotic residue analysis: LC-MS/MS methodology for ticarcillin degradation products in tomato leaves. Antibiotics 13 (2), 133. 10.3390/antibiotics13020133 38391519 PMC10886401

[B21] Hernández-García J. Briones-Moreno A. Blázquez M. A. (2021). Origin and evolution of gibberellin signaling and metabolism in plants. Semin. Cell Dev. Biol. 109, 46–54. 10.1016/j.semcdb.2020.04.009 32414681

[B22] Jha U. C. Nayyar H. Siddique K. H. (2022). Role of phytohormones in regulating heat stress acclimation in agricultural crops. J. Plant Growth Regul. 41, 1041–1064. 10.1007/s00344-021-10362-x

[B23] Jiang C. Dai J. Han H. Wang C. Zhu L. Lu C. (2020). Determination of thirteen acidic phytohormones and their analogues in tea (Camellia sinensis) leaves using ultra high performance liquid chromatography tandem mass spectrometry. J. Chromatogr. B 1149, 122144. 10.1016/j.jchromb.2020.122144 32447251

[B24] Katyayini N. U. Rinne P. L. Tarkowská D. Strnad M. van der Schoot C. (2020). Dual role of gibberellin in perennial shoot branching: inhibition and activation. Front. Plant Sci. 11, 736. 10.3389/fpls.2020.00736 32582259 PMC7289990

[B25] Kaviani B. Jamali M. Motlagh M. S. Eslami A. (2023). The effect of different levels of indole-3-butyric acid (IBA) and naphthaleneacetic acid (NAA) on the rooting of pear stem cutting. J. Hortic. Sci. 36 (4), 747–761.

[B26] Leitgeb M. Kupnik K. Knez Ž. Primožič M. (2021). Enzymatic and antimicrobial activity of biologically active samples from Aloe arborescens and Aloe barbadensis. Biology 10 (8), 765. 10.3390/biology10080765 34439997 PMC8389549

[B27] Maan A. A. Nazir A. Khan M. K. I. Ahmad T. Zia R. Murid M. (2018). The therapeutic properties and applications of Aloe vera: a review. J. Herb. Med. 12, 1–10. 10.1016/j.hermed.2018.01.002

[B28] Mangena P. (2022). Evolving role of synthetic cytokinin 6-benzyl adenine for drought stress tolerance in soybean (Glycine max L. Merr.). Front. Sustain Food Syst. 6, 992581. 10.3389/fsufs.2022.992581

[B29] Martínez-Sánchez A. López-Cañavate M. E. Guirao-Martínez J. Roca M. J. Aguayo E. (2020). Aloe vera flowers, a byproduct with great potential and wide application, depending on maturity stage. Foods 9 (11), 1542. 10.3390/foods9111542 33114533 PMC7693977

[B30] Muhammad Aslam M. Waseem M. Jakada B. H. Okal E. J. Lei Z. Saqib H. S. A. (2022). Mechanisms of abscisic acid-mediated drought stress responses in plants. Int. J. Mol. Sci. 23 (3), 1084. 10.3390/ijms23031084 35163008 PMC8835272

[B31] Mukherjee A. Gaurav A. K. Singh S. Yadav S. Bhowmick S. Abeysinghe S. (2022). The bioactive potential of phytohormones: a review. Biotechnol. Rep. 35, e00748. 10.1016/j.btre.2022.e00748 PMC9204661 35719852

[B32] Pal P. Ansari S. A. Jalil S. U. Ansari M. I. (2023). “Regulatory role of phytohormones in plant growth and development,” in Plant hormones in crop improvement (Elsevier), 1–13.

[B33] Palaniyappan S. Sridhar A. Arumugam M. Ramasamy T. (2023). Bioactive analysis of antibacterial efficacy and antioxidant potential of aloe barbadensis miller leaf extracts and exploration of secondary metabolites using GC–MS profiling. Appl. Biochem. Biotechnol. 196, 729–773. 10.1007/s12010-023-04565-z 37184725

[B34] Phytohormones as Potential Anticancer Agents (2024). Int. J. Res. Appl. Sci. Biotechnol. Available at: https://ijrasb.com/index.php/ijrasb/article/view/126 (Accessed November 27, 2024).

[B35] Pradhan B. (2023). Phytochemistry, pharmacology and toxicity of aloe vera: a versatile plant with extensive therapeutic potential. Plant Arch. 09725210 23 (2). 10.51470/plantarchives.2023.v23.no2.056

[B36] Rajesh A. Lone S. A. Ramasubburayan R. Sikkanthar S. Thajuddin N. Lee S. Y. (2023). A systemic review on Aloe vera derived natural biomaterials for wound healing applications. Biocatal. Agric. Biotechnol. 54, 102910. 10.1016/j.bcab.2023.102910

[B7] Rajmane Akash D. Shinde Komal P. (2023). A Review of HPLC Method Development and Validation as per ICH Guidelines. Asian Journal of Pharmaceutical Analysis. 13 (2), 143–151. 10.52711/2231-5675.2023.00024

[B38] Sinha L. Satyapal G. K. Kumar S. (2023). Aloe vera-A medicinal plant as potential therapeutic agents for liver cancer. Front. Med. Chem. 10 (10), 281–289. 10.2174/9789815165043123100014

[B39] Sutcharitchan C. Miao S. Li W. Liu J. Zhou H. Ma Y. (2020). High performance liquid chromatography-tandem mass spectrometry method for residue determination of 39 plant growth regulators in root and rhizome Chinese herbs. Food Chem. 322, 126766. 10.1016/j.foodchem.2020.126766 32305873

[B40] Tariq L. Bhat B. A. Hamdani S. S. (2022). “Plant growth regulators and their interaction with abiotic stress factors,” in Plant abiotic stress physiology: volume 2: molecular advancements (Apple Academic Press Inc. Palm Bay), 115–136.

[B41] Thorat J. C. Dhamal S. V. Dudheinamdar P. V. (2023). Mineral-associated medicinal plants: uncovering their anti-inflammatory potential through comprehensive exploration of bioactive compounds and pharmacological activities. J. Mines Met. Fuels 71 (11), 2095–2109. 10.18311/jmmf/2023/36273

[B42] Vaidya H. Solanki V. H. Kansara R. V. Desai C. Singh S. Patel J. (2023). Development of a novel method for multiple phytohormone analysis by UHPLC-MS/MS from bio-enriched organic fertilizer prepared using banana pseudostem sap waste. Environ. Sci. Pollut. Res. 30 (28), 71482–71490. 10.1007/s11356-022-23941-6 36376649

[B43] Wang L. Zou Y. Kaw H. Y. Wang G. Sun H. Cai L. (2020). Recent developments and emerging trends of mass spectrometric methods in plant hormone analysis: a review. Plant Methods 16 (1), 54–17. 10.1186/s13007-020-00595-4 32322293 PMC7161177

[B44] Wille S. M. R. Desharnais B. Pichini S. Trana A. D. Busardò F. P. Wissenbach D. K. (2022). Liquid chromatography high-resolution mass spectrometry in forensic toxicology: what are the specifics of method development, validation and quality assurance for comprehensive screening approaches? Curr. Pharm. Des. 28 (15), 1230–1244. 10.2174/1381612828666220526152259 35619258

[B45] Williams M. L. Olomukoro A. A. Emmons R. V. Godage N. H. Gionfriddo E. (2023). Matrix effects demystified: strategies for resolving challenges in analytical separations of complex samples. J. Sep. Sci. 46 (23), 2300571. 10.1002/jssc.202300571 37897324

[B46] Zhang M. Gao C. Xu L. Niu H. Liu Q. Huang Y. (2022). Melatonin and indole-3-acetic acid synergistically regulate plant growth and stress resistance. Cells 11 (20), 3250. 10.3390/cells11203250 36291118 PMC9600385

[B47] Zhao B. Liu Q. Wang B. Yuan F. (2021). Roles of phytohormones and their signaling pathways in leaf development and stress responses. J. Agric. Food Chem. 69 (12), 3566–3584. 10.1021/acs.jafc.0c07908 33739096

[B48] Zhong Q. Hu H. Fan B. Zhu C. Chen Z. (2021). Biosynthesis and roles of salicylic acid in balancing stress response and growth in plants. Int. J. Mol. Sci. 22 (21), 11672. 10.3390/ijms222111672 34769103 PMC8584137

