# Supplementary material for: Innovative determination of phytohormones in Aloe vera
Source: Front Chem. 2025 Jan 20;12:1490639. doi: 10.3389/fchem.2024.1490639 (PMC11788730; doi:10.3389/fchem.2024.1490639)
Supplement: Supplementary file 1 [file DataSheet1.docx]

Supplementary Information

Article

**INNOVATIVE DETERMINATION OF PHYTOHORMONES IN *ALOE VERA***

Muhammad K. Hakeem ^1^, Meera Maraqa^1^, Sampath K. Elangoven^1,2^, Esam E. Saeed^2^, Ajay K. Mishra^2^, Khaled M. Hazzouri^2^, Iltaf Shah ^1*^, Khaled M.A. Amiri^2*^

1. Department of Chemistry, College of Science, United Arab Emirates University (UAEU), Al Ain P.O. Box 15551, United Arab Emirates

2. Khalifa Center for Genetic Engineering and Biotechnology, United Arab Emirates University, Al Ain, United Arab Emirates

* **Correspondence**: altafshah@uaeu.ac.ae; k.amiri@uaeu.ac.ae

Muhammad Kamran Hakeem (MH): [700039966@uaeu.ac.ae](mailto:700039966@uaeu.ac.ae)

Meera Maraqa(MM): [202050893@uaeu.ac.ae](mailto:202050893@uaeu.ac.ae)

Sampath K Elangovan(SE): [sampath@uaeu.ac.ae](mailto:sampath@uaeu.ac.ae)

Esam Eldin Saeed(ES): [esameldin_saeed@uaeu.ac.ae](mailto:esameldin_saeed@uaeu.ac.ae)

Ajay Kumar Mishra(AM): [ajaymishra24@uaeu.ac.ae](mailto:ajaymishra24@uaeu.ac.ae)

Khaled M Hazzouri(KH): [khaled_hazzouri@uaeu.ac.ae](mailto:khaled_hazzouri@uaeu.ac.ae)

Iltaf Shah(IS): [altafshah@uaeu.ac.ae](mailto:altafshah@uaeu.ac.ae)

Khaled M.A. Amiri(KA): [k.amiri@uaeu.ac.ae](mailto:k.amiri@uaeu.ac.ae)

**LC-MS/MS Analysis and Method Development**

Q1 and Q3 in **Table 1** indicate the mass parent and mass product ion respectively. The dwell time (*Time spent acquiring specific MRM transition)* for each transition was set to 100 ms to ensure optimal data acquisition. Additionally, pre-bias values for both Q1 and Q3 ions were adjusted to fine-tune the instrument's sensitivity and stability during analysis. The ionization mode for each compound was specified **(Table 1)**, highlighting the polarity conditions under which the analysis was conducted. These optimization steps collectively contribute to the precision and accuracy of the LC-MS/MS method, ensuring robust identification and quantification of phytohormones in Aloe vera samples.

**Validation of Method**

Linearity, an essential aspect of method validation, measures the direct proportionality between detected intensity and analyte concentration in the sample. The correlation coefficient (R2) was employed to assess linearity, indicating the method's ability to produce accurate and consistent results over a specified concentration range. Matrix effect, another critical factor in validation, explores the potential deviation in the ionization mechanism and acquired data arising from sample components. The validation process serves a pivotal role in demonstrating the relevance of the analytical method for its intended purpose and ensuring the closeness of obtained values to the actual analyte content in real samples. It also enables the estimation of uncertainties associated with the measured values, enhancing the overall robustness and reliability of the LC-MS/MS analysis.

Across intraday and Interday analysis, accuracy values remained consistently high, underscoring the method's reliability in quantifying phytohormones in Aloe vera samples. For instance, ASA demonstrated robust accuracy (105.97%) at LOQ, emphasizing the method's precision even at lower concentrations. GA and I3AA exhibited comparable precision and accuracy, reaffirming the suitability of the method for quantifying these phytohormones. Additionally, 6BAP, ISA, 2NAA, and SA displayed consistent accuracy and precision across different concentration levels, further supporting the method's robustness. The relative standard deviation (RSD) and accuracy (% CV) is calculated by given formulas respectively.

$RSD=\frac{Standard deviation}{mean}$ ; $\% CV=\frac{Standard deviation}{mean}x100$

The range of linearity for the target phytohormones extends more than two orders of magnitude, with values of R^2^ of 0.99 or higher **(Table 2)**. The limit of detection (LOD) was the lowest for ISA (0.01 ng/mL) and the highest for 2NAA (36.4 ng/mL). Comparison between the results of the spiked and the observed concentrations for the Interday and intraday analysis reveals that the values fall within the acceptable accuracy range of 80%-120%. The precision in sample analysis is assessed concerning the relative standard deviation (RSD). The values of the RSD comply with the 15% acceptable margin.

**Figure S1:** Calibration curves of Individual Phytohormones

|  |  |
| --- | --- |
|  |  |
|  |  |
